# Transcriptome Analysis Reveals Intensity-Dependent Regulation of UV-B Radiation on Glucosinolate Biosynthesis in Rapeseed Leaves

**DOI:** 10.3390/plants15091335

**Published:** 2026-04-28

**Authors:** Pengpeng Mao, Song Chen, Le Kong, Xiangyu Yao, Weixuan Su, Xiaoying Liu, Yinjian Zheng, Zhigang Xu

**Affiliations:** 1 College of Agriculture, Nanjing Agricultural University, Nanjing 211800, China; mao2peng@126.com (P.M.); 2016201010@njau.edu.cn (S.C.); yaoxiangyucaas@163.com (X.Y.); 2018201024@njau.edu.cn (W.S.); 2 Rice Research Institute, Guangdong Academy of Agricultural Sciences, Guangzhou 510640, China; kongldk@163.com; 3 College of Horticulture, Nanjing Agricultural University, Nanjing 211800, China; liuxy@njau.edu.cn; 4 College of Smart Agriculture (Research Institute), Xinjiang University, Urumqi 830046, China

**Keywords:** rapeseed, glucosinolates, UV-B radiation, transcriptome, transcription factors, dose-dependent regulation

## Abstract

Rapeseed (*Brassica napus* L.) is a globally important oilseed crop; however, its ‘double-low’ cultivars exhibit substantially reduced glucosinolate levels in vegetative tissues. To investigate whether UV-B radiation could be used to enhance glucosinolate accumulation, we systematically examined the modulation of glucosinolate profiles and associated biosynthetic pathways in leaves of the ‘double-low’ cultivar NY4 under white light (WL) supplemented with two UV-B intensities: low-intensity UV-B (UVBL, 0.1 W m^−2^) and high-intensity UV-B (UVBH, 0.4 W m^−2^). Rapeseed seedlings were treated for 21 days under a 16 h photoperiod, and leaf samples were collected at the end of the treatment period, with three biological replicates per condition. Compared with the WL control, UVBL significantly increased total glucosinolate content by 64.57%, driven predominantly by elevated accumulation of progoitrin and neoglucobrassicin. In contrast, UVBH reduced total glucosinolate levels but markedly elevated gluconasturtiin content. Transcriptome analysis revealed that UVBL upregulated key genes involved in glucosinolate biosynthesis (e.g., *MAM*, *IPMDH*, *CYP79F1*, and *SOT17/18*) and transcription factors (e.g., *MYB28*, *MYB34*, *MYB51*, and *MYB122*). Conversely, UVBH downregulated genes associated with side-chain elongation of aliphatic glucosinolates and secondary modification of indolic glucosinolate. Collectively, these results demonstrate that low-intensity UV-B radiation can effectively boost total glucosinolate content in rapeseed leaves via transcriptional reprogramming.

## 1. Introduction

Rapeseed (*Brassica napus* L.) is the world’s second largest vegetable oil crop after soybean, providing 13–16% of global edible oil and serving as a key source of industrial feedstocks and biodiesel [1,2]. Glucosinolates, a group of sulfur-containing secondary metabolites, act as a key component of its defense system, which deters herbivores and pathogens through antimicrobial and antifeedant effects [3,4,5,6]. Furthermore, glucosinolates and hydrolysis products such as sulforaphane also confer human health benefits, including antioxidant, anti-inflammatory, and anticancer properties, thereby increasing interest in Brassicaceae-based functional foods [7,8,9]. Since the 1990s, ‘double-low’ varieties (low erucic acid and low seed glucosinolates) have been widely adopted to improve the safety of both oil and meal for human and animal consumption [10]. However, reduced glucosinolate levels in seeds are often correlated with decreased concentrations in vegetative tissues, rendering seedlings more susceptible to bird predation, insect infestation, and soil-borne diseases and resulting in uneven crop stands and yield instability [11]. Therefore, a central challenge in modern rapeseed production lies in decoupling vegetative defense from seed quality, which maintains robust resistance in seedlings without compromising the low-glucosinolate trait in seeds.

Current strategies to address this dilemma have focused on genetic manipulation, particularly knocking out the phloem transporters GTR1 and GTR2 to prevent the translocation of glucosinolates to seeds, thereby accumulating them in leaves [12,13]. Nevertheless, agronomic practices for dynamically modulating glucosinolate levels remain underdeveloped. Light quality is not only a key environmental factor regulating plant growth and development but also essential for modulating secondary metabolite accumulation [14,15,16]. It is widely reported that blue light preferentially enhances aliphatic glucosinolates (e.g., sinigrin, glucoraphanin, glucoerucin) by upregulating the transcription factor *MYB28* and key biosynthetic genes (e.g., *CYP79F1*, *CYP83A1*, *BCAT4*) involved in methionine chain elongation and core glucosinolate structure formation [14,17,18,19] while suppressing indolic glucosinolates such as glucobrassicin and neoglucobrassicin via downregulating pathway-specific genes (e.g., *CYP83B1*, *UGT74B1*) [20,21]. Red light promotes both aliphatic and indolic glucosinolates—for example, increasing their levels in broccoli seedlings by upregulating *SOT18* and *CYP79B2*/*B3*—via phytochrome signaling and transcription factors like HY5 and MYB28 [21,22]. In contrast, it was shown in Chinese kale sprouts that far-red light could reduce total glucosinolates, especially indolic glucosinolates, by modifying the rate of their degradation instead of the biosynthesis [23,24]. Previous study results suggest that the R–B ratio modulates two major glucosinolate classes in mustard sprouts in a species-specific manner [25]. An increased proportion of blue light within the spectrum favors the biosynthesis of aliphatic glucosinolates, while an increase in red light promotes the accumulation of indolic glucosinolates instead [25]. Moreover, green light could also synergistically enhance glucosinolates accumulation when combined with red and blue light spectra, as demonstrated in *Nasturtium officinale*, where 50% green + 35% red + 15% blue light increased total glucosinolates 5.8-fold compared to white fluorescent light [26]. While the roles of visible light are relatively clear, the full potential of the light spectrum, particularly ultraviolet radiation, remains underexplored in agronomic contexts.

Ultraviolet (UV) radiation, divided into UV-A (315–400 nm), UV-B (280–315 nm), and UV-C (100–280 nm), is a key elicitor of plant secondary metabolism [27,28]. UV-A significantly promotes both aliphatic and indolic glucosinolates via cryptochrome signaling and jasmonic acid/salicylic acid pathways [29,30], while postharvest UV-C modulates glucosinolates metabolism in a dose- and tissue-dependent manner [31]. UV-B serves as the most potent elicitor of glucosinolates accumulation in *Brassica* species, consistently enhancing aliphatic and indolic glucosinolates, such as glucoraphanin and 4-methoxyglucobrassicin [32,33,34]. However, the physiological response to UV-B is fundamentally dose- and duration-dependent. Acute or moderate UV-B typically promotes glucosinolates accumulation, while prolonged or high-intensity exposure leads to transcriptional repression of biosynthetic genes or even degradation, ultimately suppressing overall glucosinolate content [35,36]. At the molecular level, the UV-B photoreceptor UVR8-mediated signaling pathway converges on key R2R3-MYB transcription factors (e.g., MYB28 for aliphatic and MYB51 for indolic glucosinolates), which in turn activate the gene expression of core structure formations (e.g., *CYP79F1* and *CYP83B1*) and secondary modification processes (e.g., *FMOGS-OX5*) [28,32]. Notably, most evidence derives from model plants or vegetable Brassicas like broccoli and kale. The dynamic response of glucosinolate metabolism to UV-B radiation intensity in rapeseed and the comprehensive transcriptomic networks underpinning this regulation are still poorly characterized.

Therefore, to address the knowledge gaps regarding the intensity-dependent effects and molecular mechanisms of UV-B on glucosinolate metabolism in rapeseed, we conducted a systematic investigation using low-intensity (UVBL, 0.1 W m^−2^) and high-intensity (UVBH, 0.4 W m^−2^) UV-B treatments on the ‘double-low’ rapeseed cultivar NY4. Based on previous evidence, we hypothesize that (1) UV-B radiation modulates glucosinolate accumulation in rapeseed leaves in an intensity- and class-dependent manner; (2) such modulation is mediated by transcriptional reprogramming of key biosynthetic genes, with distinct expression patterns under low- and high-intensity UV-B radiation; and that (3) R2R3-MYB transcription factors play a central role in mediating the intensity-dependent regulation of glucosinolate biosynthesis pathways.

## 2. Results

### 2.1. Glucosinolate Content of Rapeseed Leaves Under Different Light Treatments

**Figure 1:**
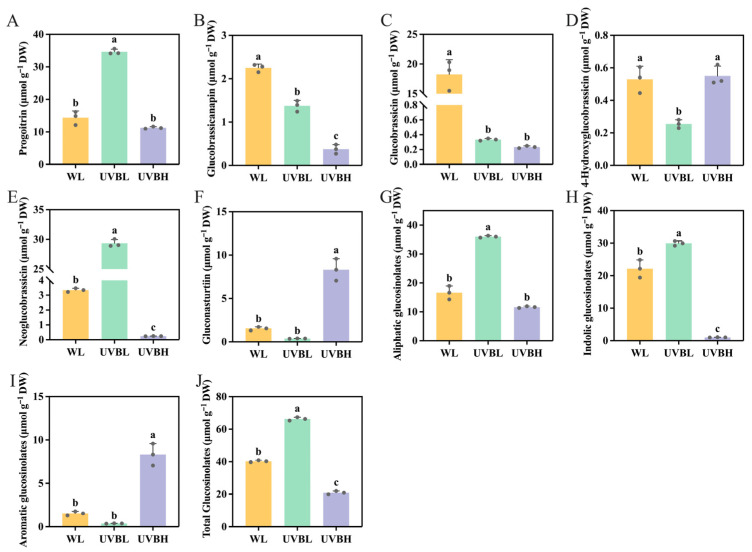
The effects of UV-B radiation on glucosinolates content in NY4 rapeseed leaves. (**A**) Progoitrin. (**B**) Glucobrassicanapin. (**C**) Glucobrassicin. (**D**) 4-Hydroxyglucobrassicin. (**E**) Neoglucobrassicin. (**F**) Gluconasturtiin. (**G**) Aliphatic glucosinolates. (**H**) Indolic glucosinolates. (**I**) Aromatic glucosinolates. (**J**) Total glucosinolates. WL: white light; UVBL: white light supplemented with 0.1 W m^−2^ UV-B radiation; UVBH: white light supplemented with 0.4 W m^−2^ UV-B radiation. Data are expressed as the average value ± SD (n = 3), and significances between treatments at *p* < 0.05 are indicated by different letters over columns.

A high-performance liquid chromatography (HPLC) analysis was conducted on NY4 rapeseed leaves exposed to three different light treatments, and the results revealed the presence of six distinct glucosinolates. These included two aliphatic glucosinolates (progoitrin and glucobrassicanapin), three indolic glucosinolates (glucobrassicin, 4-hydroxyglucobrassicin, and neoglucobrassicin), and one aromatic glucosinolate (gluconasturtiin) (Figure S3). Compared with the white light (WL) control, low-intensity UV-B radiation supplementation (UVBL, 0.1 W m^−2^) resulted in a significant increase in the contents of progoitrin (from 14.39 ± 2.26 to 34.64 ± 0.44 μmol g^−1^ DW) and neoglucobrassicin (from 3.35 ± 0.13 to 29.34 ± 0.72 μmol g^−1^ DW) while markedly reducing the levels of glucobrassicanapin, glucobrassicin, and 4-hydroxyglucobrassicin (Figure 1A–E; Table S2). In contrast, high-intensity UV-B radiation supplementation (UVBH, 0.4 W m^−2^) exerted divergent effects on individual glucosinolates. Specifically, UVBH treatment led to a significant decrease in the contents of glucobrassicanapin, glucobrassicin, and neoglucobrassicin relative to WL, but increased the content of gluconasturtiin from 1.53 ± 0.23 to 8.32 ± 1.26 μmol g^−1^ DW (Figure 1F; Table S2). Notably, there was no significant difference in the 4-hydroxyglucobrassicin content under UVBH compared to WL (Figure 1D).

UVBL treatment significantly increased the accumulation of aliphatic (by 116.47%) and indolic (by 35.23%) glucosinolates compared to WL, with no significant effect on aromatic glucosinolates (Figure 1G–I; Table S2). In contrast, UVBH treatment led to a significant reduction in indolic glucosinolates and a marked increase in aromatic glucosinolates. Furthermore, UVBL treatment significantly increased total glucosinolate levels by 64.57%, whereas UVBH treatment decreased total glucosinolate levels by 47.92% relative to the WL control (Figure 1J, Table S2). Collectively, these results suggest that UV-B radiation modulates the glucosinolate profile of NY4 rapeseed leaves in a manner that depends on both UV-B radiation intensity and glucosinolate class. Specifically, low-intensity UV-B radiation specifically promotes the accumulation of progoitrin, neoglucobrassicin, and total glucosinolates, while high-intensity UV-B radiation enhances the accumulation of aromatic glucosinolates gluconasturtiin but suppresses the accumulation of indolic glucosinolates and total glucosinolates.

### 2.2. Antioxidant Enzyme Activity of Rapeseed Leaves Under Different Light Treatments

**Figure 2:**
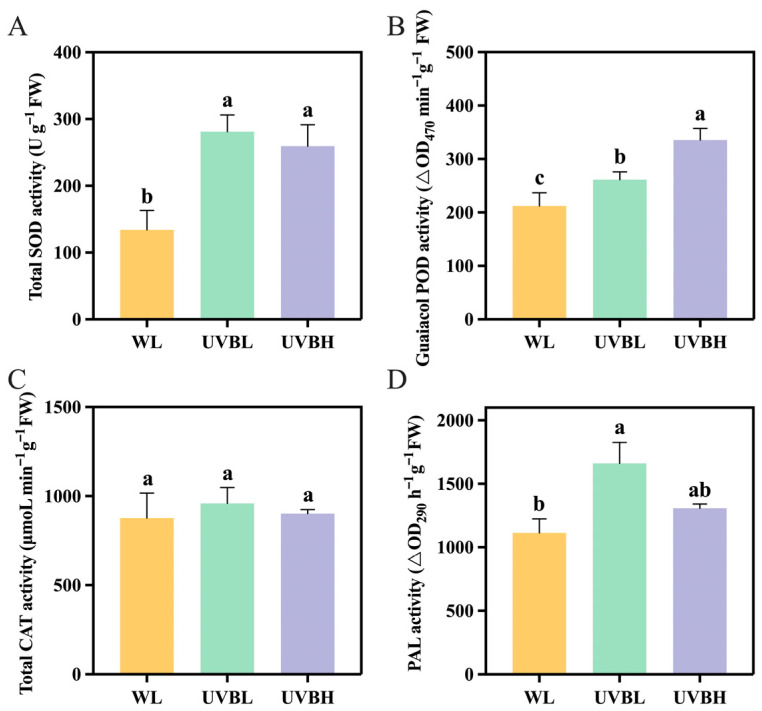
Antioxidant enzyme activity in NY4 rapeseed leaves after UV-B radiation. (**A**) Total SOD activity. (**B**) Guaiacol POD activity. (**C**) Total CAT activity. (**D**) PAL activity. SOD: superoxide dismutase; POD: peroxidase; CAT: catalase; PAL: phenylalanine ammonialyase; WL: white light; UVBL: white light supplemented with 0.1 W m^−2^ UV-B radiation; UVBH: white light supplemented with 0.4 W m^−2^ UV-B radiation. Different lowercase letters above error bars denote significant differences at *p* < 0.05.

In order to investigate the response of the antioxidant defense system in NY4 rapeseed leaves to UV-B radiation and its potential association with glucosinolate metabolism, the activities of four key enzymes—total superoxide dismutase (SOD), guaiacol peroxidase (POD), total catalase (CAT), and phenylalanine ammonialyase (PAL)—were systematically measured. The results demonstrated that, in comparison with the WL control, UV-B supplementation resulted in a significant increase in total SOD activity in rapeseed leaves (Figure 2A). Furthermore, both UVBL and UVBH treatments resulted in a marked increase in total SOD activity when compared with WL. However, no significant difference was observed between the UVBL and UVBH groups, indicating that the UV-B-induced enhancement of total SOD activity is independent of radiation intensity. The guaiacol POD activity exhibited a similar trend to that of total SOD activity, but with a potential intensity-dependent effect (Figure 2B). Specifically, guaiacol POD activity under UVBH was significantly higher than under UVBL, suggesting that high-intensity UV-B radiation exerts a more pronounced stimulatory effect on guaiacol POD activity. In contrast to total SOD and guaiacol POD activity, total CAT activity did not show a significant response to UV-B radiation (Figure 2C). In comparison with WL, UVBL treatment significantly enhanced PAL activity, resulting in the highest level observed among the three treatments, whereas UVBH did not induce a significant change in PAL activity (Figure 2D). These findings demonstrate that UV-B radiation enhances the antioxidant defense system in NY4 rapeseed leaves by selectively increasing the activities of total SOD, guaiacol POD, and PAL activity.

### 2.3. Identification of Differentially Expressed Genes (DEGs)

To elucidate the transcriptional regulatory mechanisms underlying the intensity-dependent modulation of glucosinolate biosynthesis by UV-B radiation, a transcriptome sequencing study was performed on NY4 rapeseed leaves that had been subjected to three different light treatments. Prior to conducting a differentially expressed gene (DEG) analysis, the reliability and reproducibility of the transcriptomic data were evaluated using a sample correlation heatmap and principal component analysis (PCA). As demonstrated in Figure S4, the sample correlation heatmap revealed high pairwise correlation coefficients among biological replicates within each treatment group. This finding indicates strong consistency in transcriptomic profiles across replicates. PCA further showed clear separation of the three light treatment groups along the first two principal components (PC1 and PC2), which collectively accounted for over 58.43% of the total transcriptional variation (Figure S5). The distinct clustering of samples from different treatments, as well as the tight grouping of replicates within each condition, confirm the high quality of samples and indicate that the observed transcriptional differences between groups are biologically meaningful.

**Figure 3:**
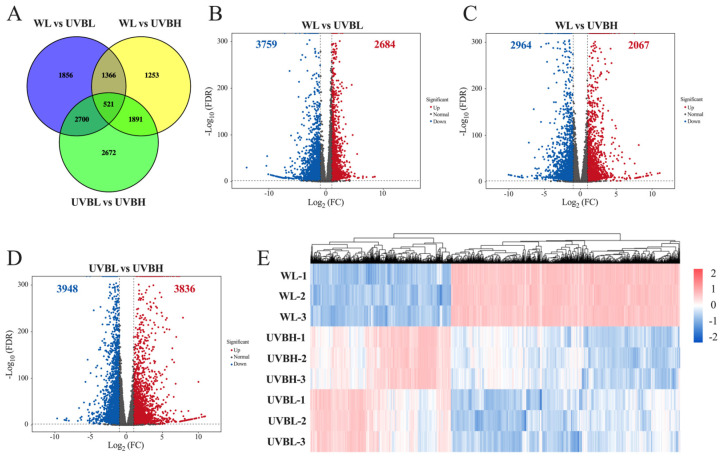
Identification of differentially expressed genes of rapeseed leaves in the different light treatments. (**A**) Venn diagram of DEGs under different comparative groups. Volcano plots of DEGs in WL vs. UVBL (**B**), WL vs. UVBH (**C**), and UVBL vs. UVBH (**D**). (**E**) Hierarchical clustering analysis on DEGs across three experimental groups. WL: white light; UVBL: white light supplemented with 0.1 W m^−2^ UV-B radiation; UVBH: white light supplemented with 0.4 W m^−2^ UV-B radiation.

DEGs were identified through pairwise comparisons (WL vs. UVBL, WL vs. UVBH, and UVBL vs. UVBH) using stringent screening criteria, and the number of DEGs varied considerably across comparison groups. In the WL vs. UVBL comparison, a total of 6443 DEGs were detected, comprising 2684 upregulated and 3759 downregulated genes (Figure 3A,B). In the comparison of WL vs. UVBH, a total of 5031 DEGs were identified, including 2067 genes that were found to be upregulated and 2964 genes that were found to be downregulated (Figure 3A,C). The comparison between UVBL and UVBH yielded the highest number of 7784 DEGs, consisting of 3836 that were found to be upregulated and 3948 that were found to be downregulated (Figure 3A,D). Across all three pairwise comparisons, a total of 12,259 unique DEGs were identified, with 521 DEGs commonly shared among all groups, suggesting a core set of transcriptional responses to UV-B radiation independent of intensity (Figure 3A). Furthermore, each comparison exhibited a substantial number of unique DEGs—1856 in WL vs. UVBL, 1253 in WL vs. UVBH, and 2672 in UVBL vs. UVBH—indicating distinct transcriptional regulatory networks activated under low- and high-intensity UV-B exposure. Hierarchical clustering analysis was performed to visualize the global expression patterns of the 12,259 DEGs across the three light treatments (Figure 3E). Genes that exhibited higher expression levels under WL generally showed reduced expression under both UVBL and UVBH, demonstrating that UV-B radiation significantly reshapes transcriptional profiles and induces pronounced expression changes relative to WL.

### 2.4. Functional Enrichment Analysis of DEGs

**Figure 4:**
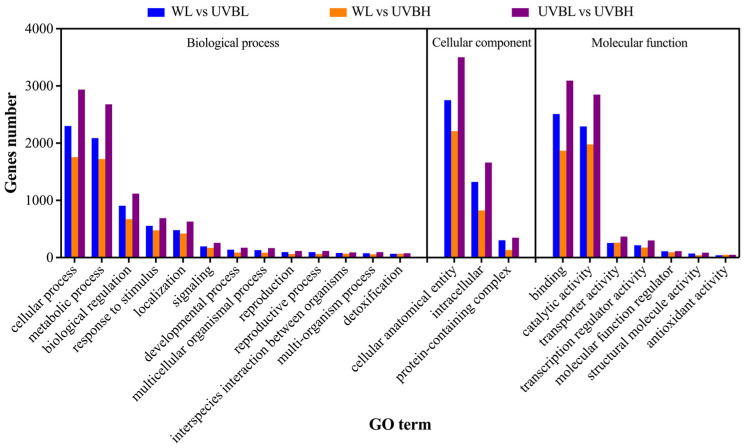
GO enrichment analysis of DEGs in the different comparative groups.

**Figure 5:**
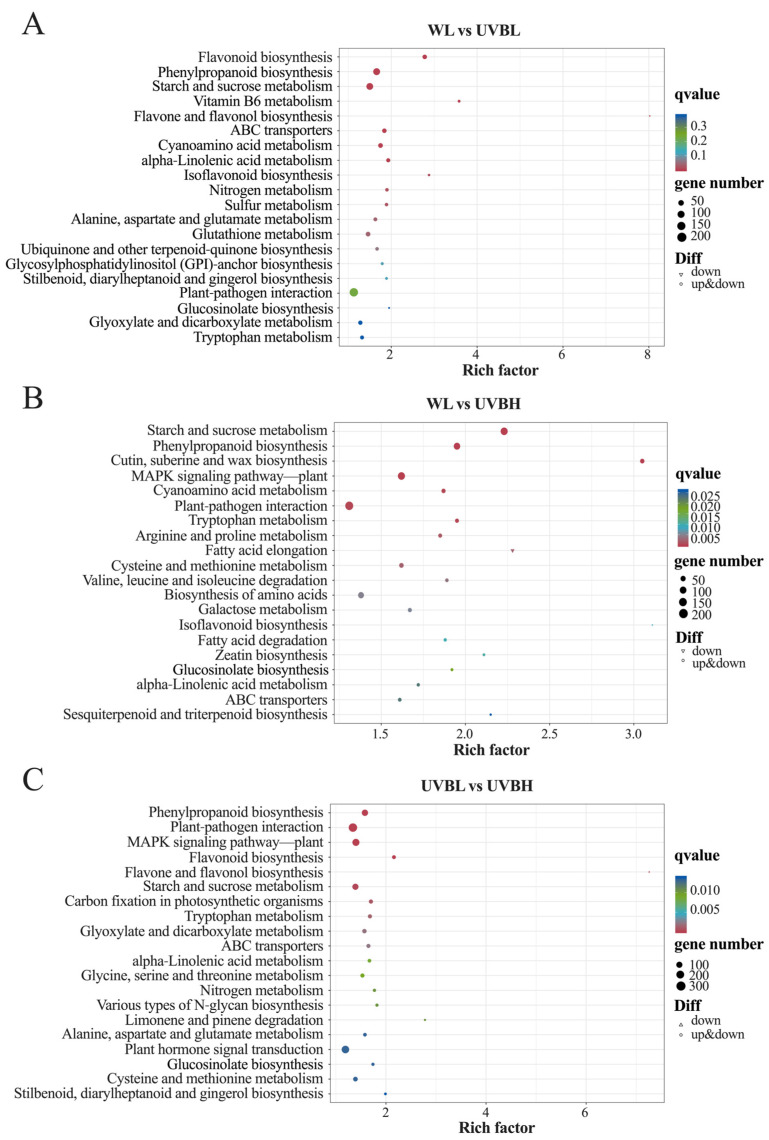
KEGG pathway enrichment analysis on DEGs in the different comparative groups. (**A**) WL vs. UVBL, (**B**) WL vs. UVBH, and (**C**) UVBL vs. UVBH.

In order to elucidate the biological functions of DEGs and their potential roles, Gene Ontology (GO) enrichment analysis and Kyoto Encyclopedia of Genes and Genomes (KEGG) pathway analysis were conducted (Figure 4 and Figure 5). GO enrichment categorized the DEGs into three core ontologies: biological process (BP), cellular component (CC), and molecular function (MF). This was undertaken to clarify the functional landscape of genes responsive to UV-B radiation (Figure 4). Compared with the WL control, a total of 5002 and 4002 DEGs in the UVBL and UVBH treatments, respectively, were successfully annotated within these three core ontologies. The most significantly enriched BP terms across the three comparison groups included “cellular process” (GO:0009987), “metabolic process” (GO:0008152), “biological regulation” (GO:0065007), and “response to stimulus” (GO:0050896). Enriched CC terms demonstrated high consistency across all three comparison groups, primarily comprising “cellular anatomical entity” (GO:0110165), “intracellular” (GO:0005622), and “protein-containing complex” (GO:0032991). MF terms commonly enriched included “binding” (GO:0005488), “catalytic activity” (GO:0003824), “transporter activity” (GO:0005215), and “transcription regulator activity” (GO:0030528). The significant enrichment of “catalytic activity” and “transcription regulator activity” indicates that UV-B radiation modulates both metabolic enzymes expression and transcriptional regulatory networks.

KEGG analysis was employed to map DEGs to known metabolic and signaling pathways, thereby unveiling pathway-specific responses to varying UV-B intensities (Figure 5A–C). In the WL vs. UVBL comparison group, significantly enriched pathways were primarily associated with secondary metabolism (Figure 5A), including “flavonoid biosynthesis” (map00941), “phenylpropanoid biosynthesis” (map00940), and “flavone and flavonol biosynthesis” (map00944). These pathways are closely linked to plant antioxidant defense systems, indicating that low-intensity UV-B radiation preferentially enhances the expression of genes involved in secondary metabolite production. In contrast, high-intensity UV-B radiation induced distinct pathway enrichment in the WL vs. UVBH comparison group (Figure 5B), with the most prominent including “starch and sucrose metabolism” (map00500), “cutin, suberine and wax biosynthesis” (map00073), and “plant–pathogen interaction” (map04626). This finding indicates that under conditions of high-intensity UV-B exposure, there is a shift in the regulatory process towards structural protection and energy reconfiguration. In the UVBL vs. UVBH comparison group, enriched pathways included “phenylpropanoid biosynthesis” (map00940), “flavone and flavonol biosynthesis” (map00944), and “plant hormone signal transduction” (map04075) (Figure 5C). The enrichment of hormone-related signaling pathways suggests that variations in UV-B intensity may modulate hormone-dependent regulatory networks, thereby driving distinct metabolic outcomes. It is noteworthy that the process of “glucosinolate biosynthesis” (map00966) was found to be consistently enriched across all three comparison groups (Figure 5A–C). This key finding directly links UV-B-induced DEGs to the core metabolic pathway of glucosinolate synthesis, confirming that UV-B radiation significantly regulates the expression of genes involved in glucosinolate biosynthesis.

### 2.5. Gene Expression Profiles in Glucosinolate Biosynthesis Pathway

**Figure 6:**
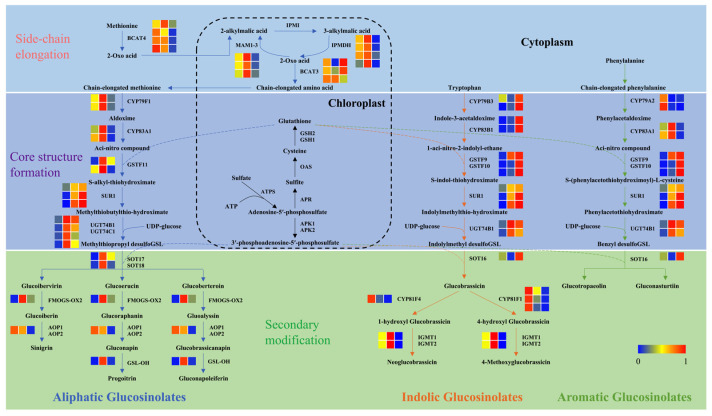
Expression levels of genes involved in the glucosinolate biosynthesis pathway identified by transcriptomic analysis. Each column in the heatmaps represents distinct homolog genes for individual enzymes, with varying colors indicating different expression levels, and from left to right, they are WL, UVBL, and UVBH. The biosynthesis pathways of all glucosinolates can be divided into three stages: side-chain elongation of amino acid precursors, core structure formation, and secondary modification. Blue, orange, and green arrows indicate the biosynthesis pathways for aliphatic, indolic, and aromatic glucosinolates, respectively.

To explore the transcriptional regulatory mechanisms underlying UV-B-dependent modulation of glucosinolate profiling, a systematic analysis of the expression patterns of key genes involved in glucosinolate biosynthesis was conducted using transcriptomic data. As illustrated in Figure 6, a heatmap is employed to visually represent the expression levels of homologous genes encoding enzymes at each catalytic step. In addition, detailed expression data (Log_2_FC values) for all 49 DEGs across three comparison groups are provided in Table S3. Glucosinolate biosynthesis is comprised of three conserved stages: amino acid precursor side-chain elongation, core structure formation, and secondary modification.

During the stage of aliphatic glucosinolate biosynthesis involving side-chain elongation, key genes exhibited distinct UV-B intensity-dependent expression patterns. In comparison with the WL control, the expression levels of *MAM1*, *MAM2*, *IPMDH2*, *IPMDH3*, *BCAT4* (*BnaC05G0396400ZS*), and *BCAT3* (*BnaC08G0298700ZS*) were found to be significantly increased under UVBL treatment but decreased under UVBH treatment. These findings suggest that low-intensity UV-B promotes this stage, whereas high-intensity UV-B inhibits it (Figure 6; Table S3).

In the core structure formation stage of aliphatic glucosinolate biosynthesis, the expression levels of *CYP79F1*, *CYP83A1*, *GSTF11*, *SUR1*, *UGT74B1*, and *UGT74C1* were higher under UVBL treatment, while *GSTF11* (*BnaA05G0489300ZS*), *SUR1*, *UGT74B1*, and *UGT74C1* were elevated under UVBH treatment compared to WL. In the process of indolic glucosinolate biosynthesis, UV-B radiation significantly increased the expression of *CYP79B3* (*BnaC04G0436200ZS*), *CYP83B1*, *GSTF9*, *GSTF10*, and *SUR1*. Furthermore, the expression levels of these genes increased progressively with increasing UV-B intensity. In the context of aromatic glucosinolate biosynthesis, the expression of *CYP83A1*, *UGT74B1*, *GSTF9*, *GSTF10*, and *SUR1* was enhanced under both UV-B treatments, whereas *CYP79A2* was downregulated.

In the secondary modification stage, a preferential upregulation of genes involved in aliphatic glucosinolate biosynthesis was observed under UV-B treatments in comparison to WL, including *SOT17*, *SOT18*, *FMOGS-OX2*, and *GSL-OH*. Notably, the highest expression levels were detected under UVBL treatment. In contrast, the expression of *AOP1* was significantly reduced following UV-B exposure. For indolic glucosinolates, UVBL treatment led to a decrease in the expression of *CYP81F1* and *CYP81F4* while increasing that of *IGMT1* and *IGMT2* relative to WL. Moreover, the expression levels of *CYP81F1*, *CYP81F4*, *IGMT1*, and *IGMT2* were found to be lowest under UVBH treatment, suggesting that high-intensity UV-B suppresses the secondary modification of indolic glucosinolates.

It is noteworthy that the expression heatmap and quantitative data across the three comparison groups are consistent with the expression patterns illustrated in the pathway diagrams (Figures S6–S8). Taken together, these results indicate that UV-B radiation modulates the expression of glucosinolate biosynthesis genes in an intensity-dependent and pathway-specific manner. Low-intensity UV-B radiation promotes the expression of key genes across all three stages of both aliphatic and indolic glucosinolate biosynthesis. Conversely, high-intensity UV-B radiation has been observed to inhibit side-chain elongation of aliphatic glucosinolates and secondary modification of indolic glucosinolates while strongly enhancing core structure formation in both indolic and aromatic glucosinolates.

### 2.6. Analysis of MYB Transcription Factor-Mediated Glucosinolate Biosynthesis Regulation

**Figure 7:**
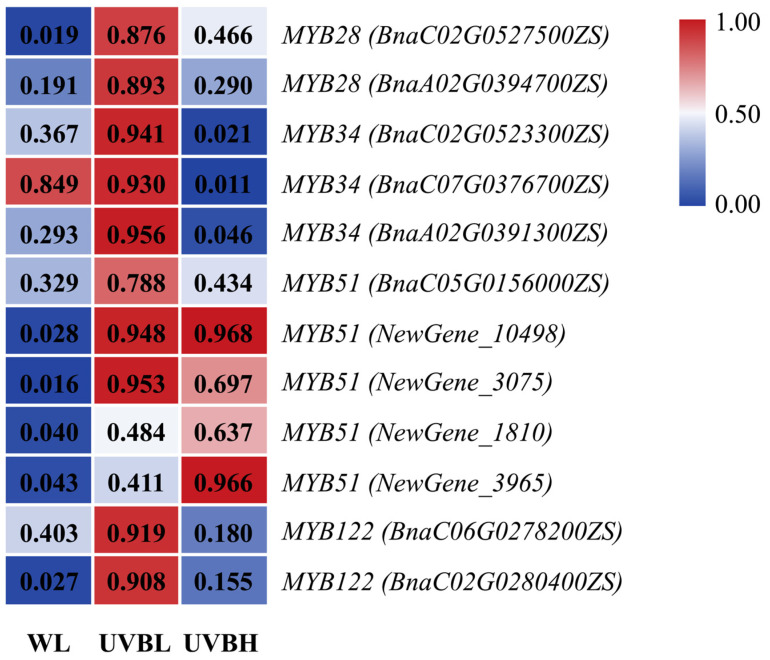
Expression levels of transcription factor genes regulating the glucosinolate biosynthesis pathway.

R2R3-MYB transcription factors have been comprehensively characterized as essential regulators of glucosinolate biosynthesis. MYB28, MYB29, and MYB76 have been shown to regulate aliphatic glucosinolate biosynthesis, whereas MYB34, MYB51, and MYB122 have been identified as key regulators of indolic and aromatic glucosinolate biosynthesis. In order to elucidate the transcriptional regulatory mechanisms underlying UV-B intensity-dependent modulation of glucosinolate profiles, a systematic analysis of the expression patterns of MYB transcription factor homologs involved in glucosinolate biosynthesis was conducted using transcriptomic data. A total of 12 differentially expressed MYB transcription factor genes, distributed across the *MYB28*, *MYB34*, *MYB51*, and *MYB122* subfamilies, were identified (Table S4). Furthermore, the expression levels of these genes have been visually summarized in a heatmap (Figure 7).

In comparison with WL, *MYB28* expression was significantly increased under UVBL, but showed no significant difference under UVBH, indicating that *MYB28* is specifically induced by low-intensity UV-B radiation. *MYB34* expression increased under UVBL but decreased under UVBH, whereas *MYB51* expression was increased under both UV-B treatments. Furthermore, *MYB122* expression was significantly increased under UVBL. Notably, the expression of *MYB28* and *MYB34* was significantly downregulated under UVBH compared to UVBL. Therefore, low-intensity UV-B radiation coordinately induces the expression of *MYB28*, *MYB34*, *MYB51*, and *MYB122*, while high-intensity UV-B selectively modulates MYB transcription factor expression, maintaining *MYB51* upregulation but suppressing *MYB28*, as well as most members of the *MYB34* and *MYB122* subfamilies. These findings demonstrate that UV-B intensity differentially regulates the expression of MYB transcription factor subfamilies, thereby mediating selective modulation of glucosinolate biosynthesis pathways.

### 2.7. Validation of EDGs by RT-qPCR

**Figure 8:**
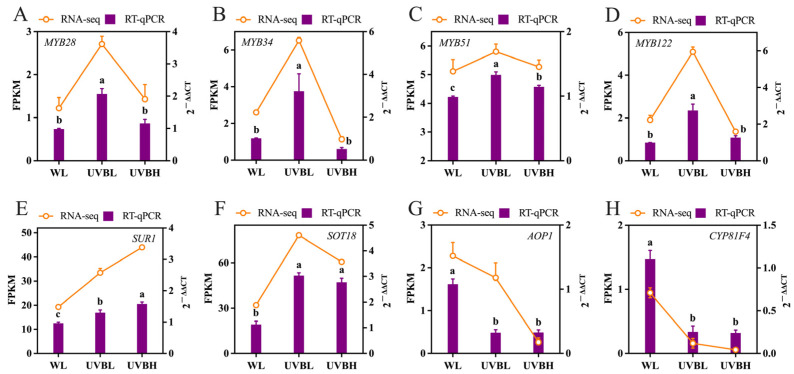
Validation of RNA-seq data using RT-qPCR. Expression levels of *MYB28* (**A**), *MYB34* (**B**), *MYB51* (**C**), *MYB122* (**D**), *SUR1* (**E**), *SOT18* (**F**), *AOP1* (**G**), and *CYP81F4* (**H**) obtained from RT-qPCR and RNA-seq. WL: white light; UVBL: white light supplemented with 0.1 W m^−2^ UV-B radiation; UVBH: white light supplemented with 0.4 W m^−2^ UV-B radiation. Values not sharing the same lowercase letter denote significant differences at *p* < 0.05.

In order to validate the accuracy and reliability of the transcriptomic data, eight key genes involved in glucosinolate biosynthesis were selected for expression analysis using reverse transcription quantitative PCR (RT-qPCR). These eight genes included four transcription factors (*MYB28*, *MYB34*, *MYB51*, and *MYB122*) and four structural genes (*SUR1*, *SOT18*, *AOP1*, and *CYP81F4*). The expression patterns obtained from RT-qPCR were then compared with those derived from the transcriptome. RT-qPCR results revealed that *MYB28*, *MYB34*, *MYB51*, and *MYB122* were significantly upregulated under UVBL compared to WL and UVBH (Figure 8A–D). For structural genes, RT-qPCR confirmed that both UVBL and UVBH significantly enhanced the expression of *SUR1* and *SOT18* while markedly suppressing the expression of *AOP1* and *CYP81F4* relative to WL (Figure 8E–H). These findings indicate that the expression trends of all eight tested genes, as measured by RT-qPCR, exhibited a high degree of consistency with the transcriptomic data.

## 3. Discussion

Rapeseed (*Brassica napus* L.) is the world’s second largest vegetable oil crop, and ‘double-low’ varieties with low erucic acid and low seed glucosinolates have been widely adopted to ensure food and feed safety [2,37]. However, the reduced glucosinolate content in the vegetative tissues of these varieties leads to poor seedling resistance to herbivores, insects, and pathogens, thereby threatening crop establishment and yield stability [2,11]. A key challenge in rapeseed breeding remains the decoupling of vegetative defense from seed quality, which involves the maintenance of high glucosinolate levels in seedlings while preserving low glucosinolate content in seeds. Light quality is a crucial environmental factor regulating plant secondary metabolism. Indeed, UV-B radiation has been demonstrated to potently induce glucosinolate accumulation in *Brassica* species [16,32,34]. Nevertheless, the intensity-dependent effects and underlying molecular mechanisms of UV-B on glucosinolate biosynthesis in rapeseed are still poorly understood. To address these knowledge gaps, this study systematically investigated the impacts of low-intensity (UVBL, 0.1 W m^−2^) and high-intensity (UVBH, 0.4 W m^−2^) UV-B radiation on glucosinolate profiles and biosynthetic pathways in NY4 rapeseed leaves using HPLC analysis and transcriptome sequencing (Figures S1 and S2).

In this study, UV-B radiation exerted distinct effects on glucosinolate classes and individual components in rapeseed leaves, with a clear dependence on intensity (Figure 1). The application of low-intensity UV-B radiation resulted in a substantial increase in total glucosinolate content, amounting to 64.57%. This enhancement was predominantly attributed to the promotion of aliphatic (progoitrin) and indolic (neoglucobrassicin) glucosinolates, as elucidated in Table S2. In contrast, exposure to high-intensity UV-B radiation resulted in a decrease in total glucosinolates; however, it selectively increased the levels of aromatic glucosinolates, specifically gluconasturtiin. As previously reported, moderate UV-B (0.6 kJ m^−2^ d^−1^) was found to significantly increase aliphatic (4-methylsulfinylbutyl glucosinolate) and indolic (4-methoxyindol-3-ylmethyl glucosinolate) glucosinolates in broccoli sprouts, with total glucosinolates increasing by approximately 42% [32]. Similarly, periodic low-dose UV-B treatment increased total glucosinolates by 40% in kale sprouts, with specific increases in glucoraphanin and 4-hydroxy-indolylglucosinolate [35]. In contrast, a study on *Arabidopsis* found that prolonged exposure to high-intensity UV-B (1.55 W m^−2^) for 12 h reduced total glucosinolates, particularly indolic glucosinolates [36]. This finding is consistent with the suppression of indolic glucosinolates under high-intensity UV-B observed in this study. It is noteworthy that the selective induction of aromatic glucosinolates by high-intensity UV-B is a significant finding in rapeseed. UV-B exposure has been observed to enhance aliphatic glucosinolates in radish sprouts (accounting for 85% of the total) and indolic glucosinolates in broccoli sprouts; however, no enrichment of aromatic glucosinolates was observed [38]. This species-specific response highlights that rapeseed employs a distinct glucosinolate remodeling strategy in response to high-intensity UV-B, thereby expanding the current understanding of UV-B-induced glucosinolate metabolism in Brassicaceae.

The intensity-dependent changes in glucosinolate profiles were closely associated with the transcriptional regulation of biosynthetic genes (Figure 3, Figure 4, Figure 5 and Figure 6; Table S3). Low-intensity UV-B was found to upregulate key genes involved in all three stages of glucosinolate biosynthesis: side-chain elongation (*MAM1/2*, *IPMDH2/3*), core structure formation (*CYP79F1*, *CYP83A1*), and secondary modification (*SOT17/18*, *IGMT1/2*). In contrast, high-intensity UV-B radiation led to the downregulation of genes associated with side-chain elongation (*MAM1*, *IPMDH3*) and indolic glucosinolate secondary modification (*CYP81F1*, *IGMT1/2*) while concurrently enhancing those involved in core structure formation (*CYP79B3*, *GSTF10*). These findings are consistent with previously reported molecular mechanisms. UV-B regulates glucosinolate biosynthesis via the UVR8-COP1-HY5 signaling pathway, which activates downstream biosynthetic genes [34]. It was demonstrated that prolonged exposure to UV-B radiation in pakchoi could enhance the expression of glucosinolate biosynthesis-related genes, a process that is facilitated by JA/SA signaling pathways [27]. Furthermore, studies have shown that blue light upregulates *CYP79F1* and *CYP83A1*, thereby promoting the accumulation of aliphatic glucosinolates in broccoli sprouts, which suggested the presence of a conserved regulatory mechanism for core biosynthetic genes across different light signals [19]. The present study extends these insights by demonstrating that UV-B intensity specifically modulates genes in the side-chain elongation and secondary modification pathways. This provides a mechanistic explanation for the differential accumulation of glucosinolate classes. Additionally, UV-A has been observed to upregulate *BCAT4* and *MAM1* in Chinese kale, thereby underscoring the conserved role of UV radiation in activating side-chain elongation genes. However, the intensity-dependent suppression observed in this study unveils an additional layer of regulatory complexity in rapeseed [29].

R2R3-MYB transcription factors have been shown to play a central role in mediating the dose-dependent regulation of glucosinolate biosynthesis [39,40,41,42]. Low-intensity UV-B radiation coordinately upregulated *MYB28*, *MYB34*, *MYB51*, and *MYB122*, whereas high-intensity UV-B radiation has been observed to maintain only the upregulation of *MYB51* while suppressing *MYB28*, *MYB34*, and *MYB122* (Figure 7; Table S4). These findings are consistent with the established functions of MYB transcription factors. A more comprehensive overview is provided by MYB28, MYB29, and MYB76, which function as specific regulators of aliphatic glucosinolates, and MYB34, MYB51, and MYB122, which act as pivotal regulators of indolic glucosinolates [43,44]. UV-B enhances the melatonin-induced expression of *MYB28.2* and *MYB51.2* in Chinese kale sprouts, confirming the involvement of these transcription factors in UV-B signaling pathways [28]. The present study demonstrated that the differential regulation of distinct MYB subfamilies by varying UV-B intensities resulted in the activation of *MYB28* under low UV-B radiation, thereby promoting aliphatic glucosinolate biosynthesis. In contrast, sustained *MYB51* expression under high UV-B radiation was found to contribute to aromatic glucosinolate biosynthesis. RT-qPCR validation of eight key genes confirmed the reliability of the transcriptomic data (Figure 8).

**Figure 9:**
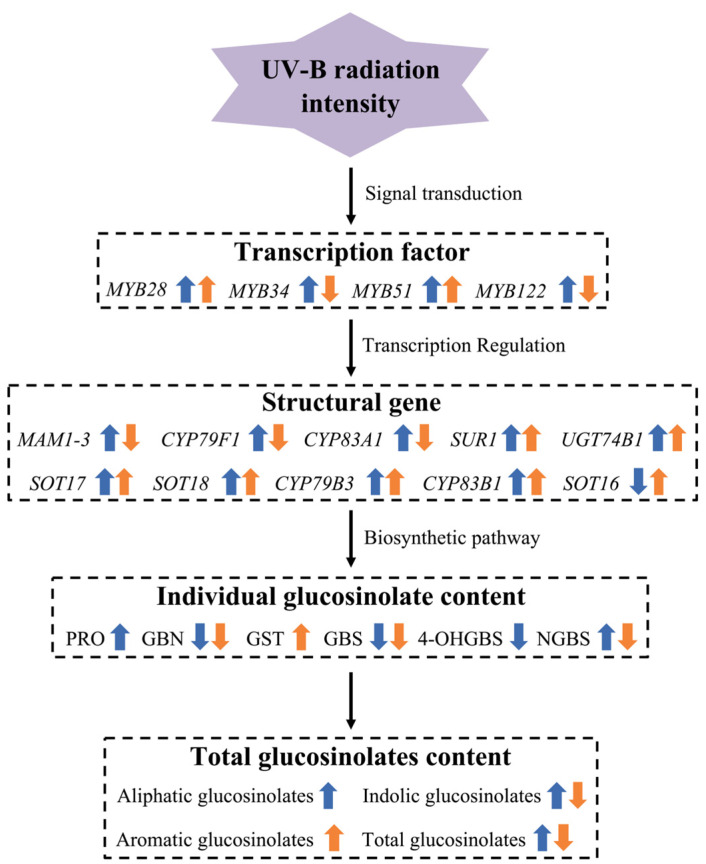
Schematic diagram of UV-B radiation intensity-dependent regulation on glucosinolate biosynthesis. Blue and yellow arrows indicate the effects of low-intensity and high-intensity UV-B radiation on gene expression levels of the glucosinolate biosynthesis pathway and glucosinolate content, respectively. An upward arrow represents promotion or increase, while a downward arrow represents inhibition or decrease.

The regulatory mechanism by which UV-B intensity influences glucosinolate biosynthesis in rapeseed likely involves the integration of light signaling, transcriptional regulation, and stress responses (Figure 9). The perception of low-intensity UV-B radiation is facilitated by the UVR8 photoreceptor, which undergoes a dissociation process to form individual monomers, subsequently engaging with COP1 to regulate the stability of the HY5 transcription factor [34,45]. HY5 subsequently activates *MYB28*, *MYB34*, *MYB51*, and *MYB122*, which upregulate genes involved in all stages of glucosinolate biosynthesis, resulting in increased accumulation of both aliphatic and indolic glucosinolates. In contrast, high-intensity UV-B may induce an excessive production of reactive oxygen species, which have been shown to suppress genes associated with side-chain elongation and indolic secondary modifications [36]. In addition, high-intensity UV-B has been demonstrated to activate the expression of core structure genes involved in aromatic glucosinolate synthesis, potentially through JA/SA-mediated stress signaling pathways [27]. Furthermore, the UV-B-induced increases in total SOD, guaiacol POD, and PAL activities observed in this study suggest that the antioxidant defense system is synergistically activated alongside glucosinolate metabolism, forming a coordinated defense network against UV-B stress.

This study aims to addresses the existing knowledge gap regarding the intensity-dependent regulatory mechanisms of UV-B radiation on glucosinolate metabolism in rapeseed. The findings presented herein provide a theoretical foundation for the development of novel breeding strategies to enhance stress resistance in ‘double-low’ rapeseed varieties. Specifically, the identification of key regulatory genes and transcription factors that are responsive to UV-B radiation offers potential molecular targets for marker-assisted selection or gene editing approaches. These approaches have the potential to engineer rapeseed varieties with enhanced glucosinolate biosynthesis in vegetative tissues while maintaining low seed glucosinolate content. Nevertheless, this study is subject to several limitations that necessitate further investigation. Firstly, the investigation was conducted using the NY4 rapeseed cultivar exclusively. It is possible that intensity-dependent regulatory patterns vary among cultivars; therefore, further studies involving diverse ‘double-low’ varieties are required to validate the generalizability of the findings. Secondly, the duration of UV-B exposure was not examined. A previous study reported that short-term UV-B exposure promoted flavonoid accumulation, whereas long-term exposure shifted metabolism towards glucosinolate accumulation in pakchoi [27]. This finding indicates that further research is required to clarify the interaction between exposure duration and intensity. Thirdly, the role of UVR8 and downstream signaling hormones (e.g., JA, SA) in mediating UV-B-induced responses was not directly verified. Future studies employing UVR8 mutants or hormone inhibitors may facilitate the elucidation of the underlying signaling cascade.

## 4. Materials and Methods

### 4.1. Plant Materials and Growth Conditions

Approximately 300 seeds of rapeseed (*Brassica napus* L., cv. NY4, provided by Nanjing Agricultural University) were uniformly sown in culture dishes (16 mm in height, 142 mm in bottom diameter; Changde Bkmam Biotechnology Co., Ltd., Changde, China) lined with two layers of pre-moistened absorbent paper under the bottom, which is described in previous study [37]. The seeds in the culture dishes were then subjected to germination under different light treatments in an artificial climate chamber: white light (WL), white light supplemented with low-intensity UV-B radiation (UVBL), and high-intensity UV-B radiation (UVBH). The photosynthetic photon flux density of WL at 30 cm above the culture dishes was maintained at 200 μmol m^−2^ s^−1^, while the intensity of UVBL and UVBH was set at 0.1 and 0.4 W m^−2^, respectively (measured using an OHSP-350UV spectrometer; Hangzhou Hopoo Light & Color Technology Co., Ltd., Hangzhou, China) (Figure S1).

The artificial climate chamber was maintained under a 16 h photoperiod, a day/night temperature regime of 25 °C/18 °C, and a relative humidity of (70 ± 10%). During the germination period, 5 mL of Hoagland’s nutrient solution (pH 6.0) was added to each culture dish twice daily until the cotyledons fully expanded, which occurred 7 days after sowing. Subsequently, uniformly growing seedlings were selected and transplanted into 32-cell plug trays (cell dimensions: 5.9 cm upper diameter, 3.8 cm bottom diameter, 6.2 cm depth; external tray size: 54 cm × 28 cm). The growth substrate consisted of a mixture of peat, vermiculite, and perlite at a volume ratio of 3:1:1 (*v*/*v*/*v*), which was sterilized by autoclaving at 121 °C for 30 min prior to use. The growth conditions for the transplanted seedlings remained identical to those during germination. Every three days, 200 mL of Hoagland’s nutrient solution was supplied to each tray. Based on preliminary results from previous studies on UV-B exposure durations, leaf samples were collected from at least 24 plants at 21 days after sowing (corresponding to the three true-leaf stage) for subsequent analyses (Figure S2). All collected samples were immediately flash-frozen in liquid nitrogen and stored at −80 °C until further processing.

### 4.2. Glucosinolate Profile Measurements by HPLC

Glucosinolates were extracted and analyzed as previously described in ISO 9167:2019 [46], with slight modifications. Briefly, 0.10 g of freeze-dried leaf samples were pre-heated in a 90 °C water bath for 1 min, followed by mixing with 4 mL of 70% methanol and 200 μL of 5 mM sinigrin internal standard. After incubation at 75 °C for 20 min with shaking and then centrifugation at 5000× *g* for 10 min, the supernatant was collected. The residue was re-extracted with 2 mL of 70% methanol after a 75 °C water bath for 10 min. After centrifugation, collect the supernatant and combine it with the previous supernatant.

The column packed with 0.5 mL of DEAE-Sephadex A-25 suspension pre-equilibrated in 2 mol L^−1^ of acetic acid was sequentially rinsed with 2 mL of 6 M imidazole formate and twice with 1 mL of ddH_2_O. A total of 1 mL of combined supernatants were loaded onto the column and washed twice with 1 mL of 0.02 M sodium acetate buffer (pH 4.0). Glucosinolates were enzymatically desulfated at ambient temperature for approximately 15 h with 100 μL of diluted purified sulfatase (EC 3.1.6.1). The resulting desulfoglucosinolates were eluted with 1 mL of ddH_2_O. Eluates were filtered through a 0.22 μm PTFE filter.

High-performance liquid chromatography (HPLC) analysis of desulfoglucosinolates was performed using a Shimadzu HPLC system (Shimadzu, Kyoto, Japan) equipped with an SPD-M20A diode array detector. A 20 μL aliquot of each sample was injected on a Symmetry C18 column (5 μm particle size, 4.6 mm × 250 mm; Elite Analytical Instruments Co., Ltd., Dalian, China) using a gradient mobile phase consisting of ultrapure water (eluent A) and acetonitrile (eluent B) at a flow rate of 1.0 mL min^−1^. The gradient program was as follows: 0–38 min, 0% B to 20% B; 38–40 min, hold at 20% B; 40–45 min, increase from 20% B to 100% B; 45–50 min, decrease from 100% B to 0% B; and 50–60 min, hold at 0% B. The column temperature was 30 °C, and the detector wavelength was 229 nm. Sinigrin (Sigma) was used as an internal standard for HPLC quantification. The chromatographic profile of glucosinolates is shown in Figure 3A, and the corresponding trivial names and abbreviations are provided in Figure 3B.

### 4.3. Measurement of Antioxidant Enzyme Activity

Fresh leaf samples (0.10 g) were homogenized in ice-cold phosphate buffer (50 mM, pH 7.8) and subsequently centrifuged at 12,000× *g* for 15 min. The supernatant was collected and used for enzyme activity assays, which were performed using commercial kits (Suzhou Geruisi Biotechnology Co., Ltd., Suzhou, China). Guaiacol peroxidase (POD, EC 1.11.1.7) activity was determined by measuring the oxidation of guaiacol at 470 nm. Total catalase (CAT, EC 1.11.1.6) activity was assessed by monitoring the decomposition of H_2_O_2_ at 240 nm. Phenylalanine ammonialyase (PAL, EC 4.3.1.24) activity was quantified based on the catalytic cleavage of L-phenylalanine at 290 nm. Total superoxide dismutase (SOD, EC 1.15.1.1) activity was measured by tracking the color change of the nitroblue tetrazolium (NBT) solution resulting from its reaction with superoxide anions at 560 nm. All reagents, samples, and standards were prepared according to the manufacturer’s instructions. Enzyme activities were calculated according to the guidelines provided in the assay kits. Three biological replicates were analyzed for each treatment.

### 4.4. RNA Extraction and Transcriptome Sequencing

Total RNA was extracted from rapeseed leaf tissues using the RNAprep Pure Plant Kit (Tiangen, Beijing, China) according to the manufacturer’s instructions. Three individual plants were pooled to constitute one biological sample, and three such samples in each treatment were prepared. RNA concentration, integrity, and purity were assessed using a NanoDrop 2000 spectrophotometer (Thermo Fisher Scientific, Wilmington, DE, USA) and the RNA Nano 6000 Assay Kit on the Agilent Bioanalyzer 2100 system (Agilent Technologies, Santa Clara, CA, USA).

For library preparation, 1 μg of total RNA per sample was used as input material. Sequencing libraries were generated using the NEBNext Ultra^TM^ RNA Library Prep Kit for Illumina (NEB, Ipswich, MA, USA), following the manufacturer’s protocol, with unique index codes added to each sample for multiplexing. The resulting libraries were sequenced on an Illumina HiSeq 2500 platform by Biomarker Technologies Co., Ltd. (Beijing, China), generating paired-end reads. Raw reads were filtered to remove low-quality sequences and adaptors, and the resulting clean reads were mapped and quantified. Reads with either a perfect match or no more than one mismatch were aligned to the ZS11 reference genome (http://cbi.hzau.edu.cn/bnapus/) (accessed on 14 April 2025) by HISAT2. Transcript abundance was estimated using the FPKM (fragments per kilobase of transcript per million base pairs) method. Differentially expressed genes (DEGs) were identified through DESeq2, with an adjusted *p*-value < 0.05 and an absolute log2 foldchange > 1 as thresholds.

### 4.5. Reverse Transcription Quantitative PCR

Total RNA was extracted from rapeseed leaf tissues using TRIzol™ reagent (Invitrogen) according to the manufacturer’s instructions. The extraction was performed in triplicate for each biological sample. RNA concentration and purity (A_260_/A_280_ ratio) were assessed using a ScanDrop 100 spectrophotometer (YOMIM, Hangzhou, China). First-strand cDNA was synthesized from qualified RNA using the TUREscript 1st Strand cDNA Synthesis Kit (Aidlab, Beijing, China), following the manufacturer’s protocol.

Reverse transcription quantitative polymerase chain reaction (RT-qPCR) was carried out on a qTOWER2.2 instrument (Analytik Jena, Jena, Thuringia, Germany). Each 10 µL reaction mixture contained 1 µL of cDNA template, 0.5 µL of each gene-specific primer (final concentration: 200 nM), 5 µL of 2× SYBR Green Supermix (DBI), and 3 µL of nuclease-free ddH_2_O. The amplification program consisted of an initial denaturation at 95 °C for 3 min, followed by 39 cycles of 95 °C for 10 s and 58 °C for 30 s. The *Actin7* gene was used as the endogenous reference gene for data normalization. Gene expression levels were calculated using the 2^−ΔΔCT^ method. All treatments and controls were analyzed with three biological replicates and three technical replicates. Primer sequences are listed in Table S1.

### 4.6. Statistical Analysis

All measurements for each treatment in this study were performed using at least three independent biological replicates. For the heatmap, the data were normalized using z-scores and visualized using the heatmap package. All data were processed and analyzed with IBM SPSS software (version 25.0, IBM, New York, NY, USA). Duncan’s multiple range test was applied to determine significant differences among treatments at the *p* < 0.05 level.

## 5. Conclusions

This study systematically investigated the regulatory effects of UV-B radiation at different intensities (UVBL: 0.1 W m^−2^; UVBH: 0.4 W m^−2^) on glucosinolate biosynthesis in leaves of ‘double-low’ rapeseed (*Brassica napus* L. cv. NY4) using HPLC-based metabolic profiling and transcriptome sequencing. The results clearly demonstrate that UV-B radiation modulates glucosinolate accumulation in a dose-dependent and class-specific manner. UVBL significantly increased total glucosinolate content by 64.57% compared to the white light (WL) control, primarily by enhancing aliphatic (e.g., progoitrin) and indolic (e.g., neoglucobrassicin) glucosinolates. In contrast, UVBH reduced total glucosinolate levels by 47.92%, but selectively increased the aromatic glucosinolate gluconasturtiin.

The intensity-dependent modulation of glucosinolate profiles is mediated by the transcriptional reprogramming of key biosynthetic genes and R2R3-MYB transcription factors. UVBL upregulated genes involved in all three stages of glucosinolate biosynthesis (side-chain elongation, core structure formation, and secondary modification) and coordinately activated *MYB28*, *MYB34*, *MYB51*, and *MYB122*. In contrast, UVBH was observed to exert a suppressive effect on genes associated with side-chain elongation of aliphatic glucosinolate and secondary modification of indolic glucosinolate while enhancing the expression of core structure formation genes and maintaining the upregulation of *MYB51*. Furthermore, UV-B radiation synergistically activated the antioxidant defense system, selectively increasing the activities of total SOD, guaiacol POD, and PAL, thereby establishing a coordinated stress response network.

In summary, the present study aims to address the existing knowledge gap concerning the intensity-dependent regulatory mechanisms of UV-B radiation on glucosinolate metabolism in rapeseed. The findings presented herein provide a theoretical foundation for the development of novel breeding strategies, with the goal of enhancing the stress resistance of ‘double-low’ rapeseed varieties.

## Data Availability

The data provided in this study are available from the corresponding author upon reasonable request. The data are not publicly available due to privacy.

## Supplementary Materials

The following supporting information can be downloaded at https://www.mdpi.com/article/10.3390/plants15091335/s1. Figure S1: Spectrum diagram in this experiment; Figure S2: UV-B treatments and sample process; Figure S3: The glucosinolate chromatogram and name; Figure S4: Sample correlation heatmap; Figure S5: Principal component analysis plot; Figure S6: Glucosinolate biosynthesis of KEGG pathway enrichment analysis on DEGs in WL vs. UVBL; Figure S7: Glucosinolate biosynthesis of KEGG pathway enrichment analysis on DEGs in WL vs. UVBH; Figure S8: Glucosinolate biosynthesis of KEGG pathway enrichment analysis on DEGs in UVBL vs. UVBH; Table S1: The primer sequences used in this RT-qPCR assay; Table S2: Glucosinolate content of oilseed rape leaves in different light treatments; Table S3: The expression of DEGs involved in glucosinolate biosynthesis; Table S4: DEGs of transcription factor regulated glucosinolate biosynthesis.

## Abbreviations

The following abbreviations are used in this manuscript:

UV	Ultraviolet
WL	White light
HPLC	High-performance liquid chromatography
POD	Peroxidase
CAT	Catalase
PAL	Phenylalanine ammonialyase
SOD	Superoxide dismutase
NBT	Nitroblue tetrazolium
FPKM	Fragments per kilobase of transcript per million base pairs
DEGs	Differentially expressed genes
RT-qPCR	Reverse transcription quantitative polymerase chain reaction
GO	Gene Ontology
KEGG	Kyoto Encyclopedia of Genes and Genomes
PCA	Principal component analysis
