# Supplementary material for: Transcriptome Analysis Reveals Intensity-Dependent Regulation of UV-B Radiation on Glucosinolate Biosynthesis in Rapeseed Leaves
Source: Plants (Basel). 2026 Apr 28;15(9):1335. doi: 10.3390/plants15091335 (PMC13164901; doi:10.3390/plants15091335)
Supplement: Supplementary file 1 [file plants-15-01335-s001.zip › plants-4126221-supplementary.pdf]

Supplementary Materials

Transcriptome analysis reveals intensity-dependent regulation of UV-B radiation on glucosinolate biosynthesis in rapeseed leaves

Pengpeng Mao [1†], Song Chen [1†], Le Kong [3], Xiangyu Yao [1], Weixuan Su [1], Xiaoying Liu [4], Yinjian Zheng [2*], Zhigang Xu [1*]

[1] *College of Agriculture, Nanjing Agricultural University, Nanjing 211800, China*

[2] *College of Smart Agriculture (Research Institute), Xinjiang University, Urumqi 830046, China*

[3] *Rice Research Institute, Guangdong Academy of Agricultural Sciences, Guangzhou 510640, China*

[4] *College of Horticulture, Nanjing Agricultural University, Nanjing 211800, China*

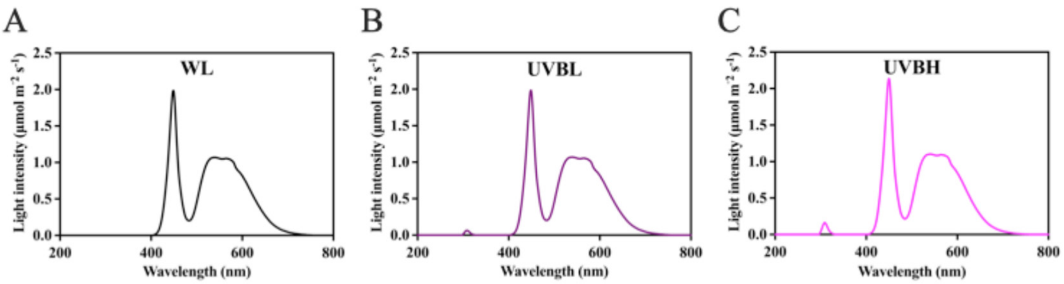

Figure S1. Spectrum diagram in this experiment. **(A)** WL. **(B)** UVBL. **(C)** UVBH. WL: white light, UVBL: white light supplemented with 0.1 W m^{-2} UV-B radiation, UVBH: white light supplemented with 0.4 W m^{-2} UV-B radiation.

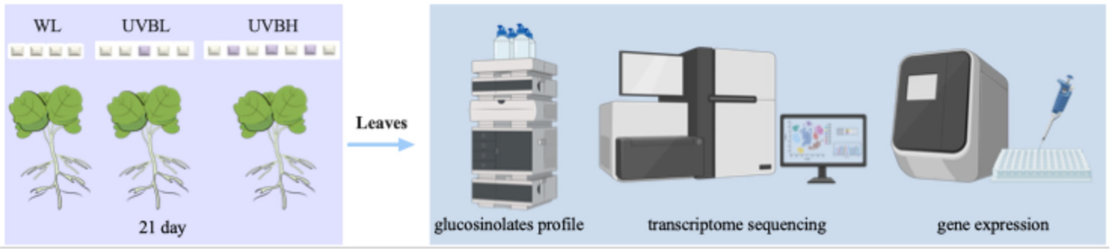

Figure S2. UV-B treatments and sample process. WL: white light, UVBL: white light supplemented with 0.1 W m^{-2} UV-B radiation, UVBH: white light supplemented with 0.4 W m^{-2} UV-B radiation.

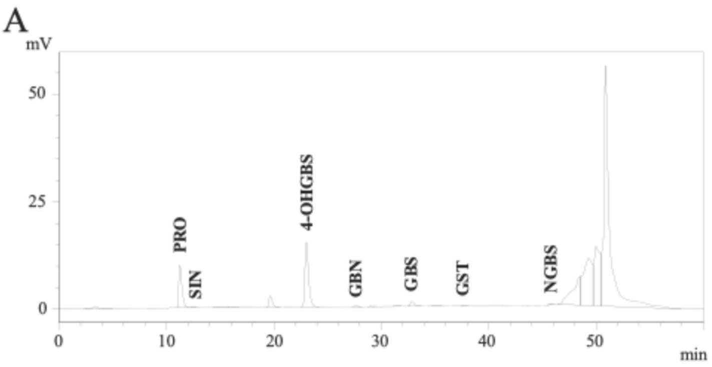

B

Abbreviation	Trivial name
**Aliphatic glucosinolates**	
PRO	Progoitrin
SIN	Sinigrin
GBN	Glucobrassicanapin
**Indolic glucosinolates**	
GBS	Glucobrassicin
4-OHGBS	4-Hydroxyglucobrassicin
NGBS	Neoglucobrassicin
**Aromatic glucosinolates**	
GST	Gluconasturtiin

Figure S3. The glucosinolate chromatogram and name. **(A)** Glucosinolate chromatogram. **(B)** The trivial name and abbreviation of glucosinolates.

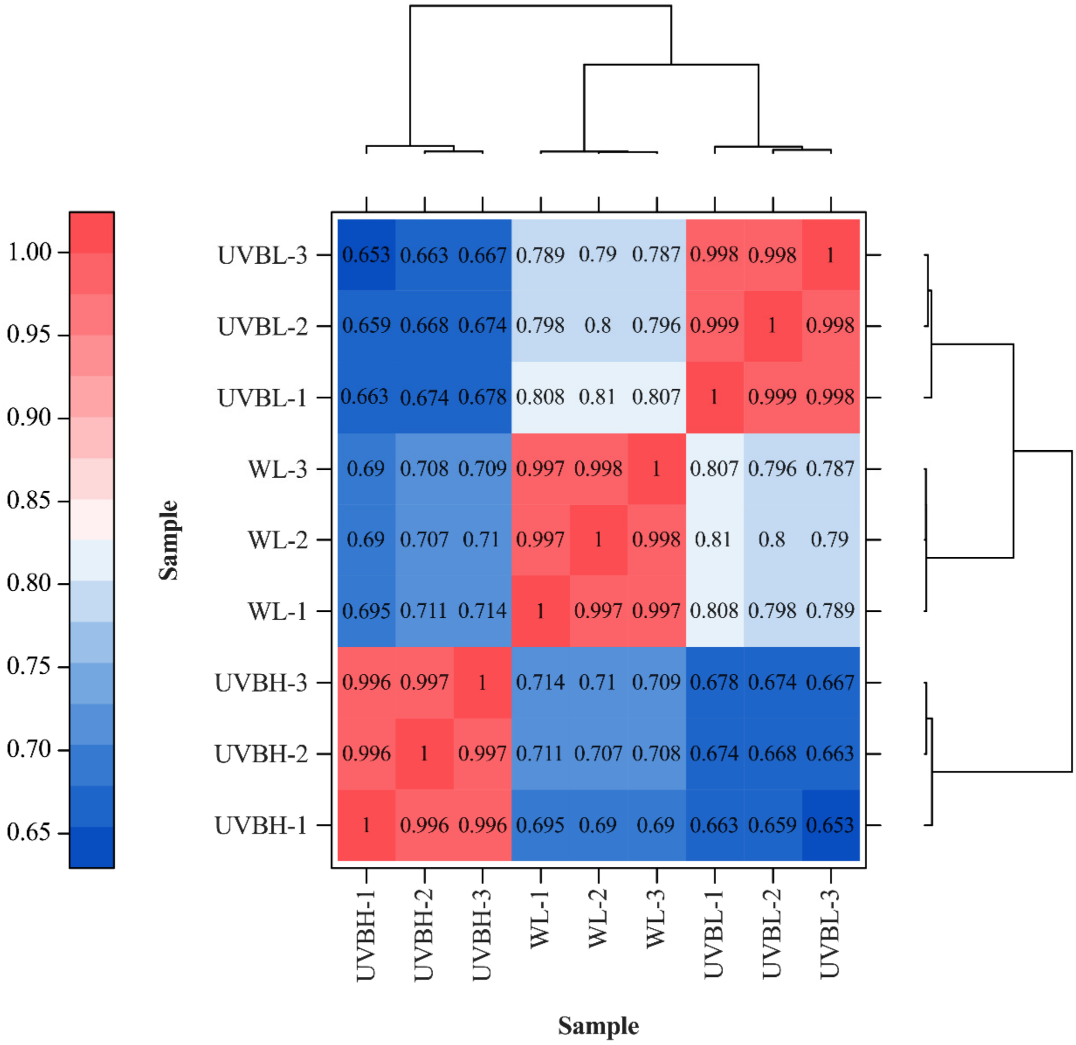

Figure S4. Sample correlation heatmap. WL: white light, UVBL: white light supplemented with 0.1 W m^{-2} UV-B radiation, UVBH: white light supplemented with 0.4 W m^{-2} UV-B radiation.

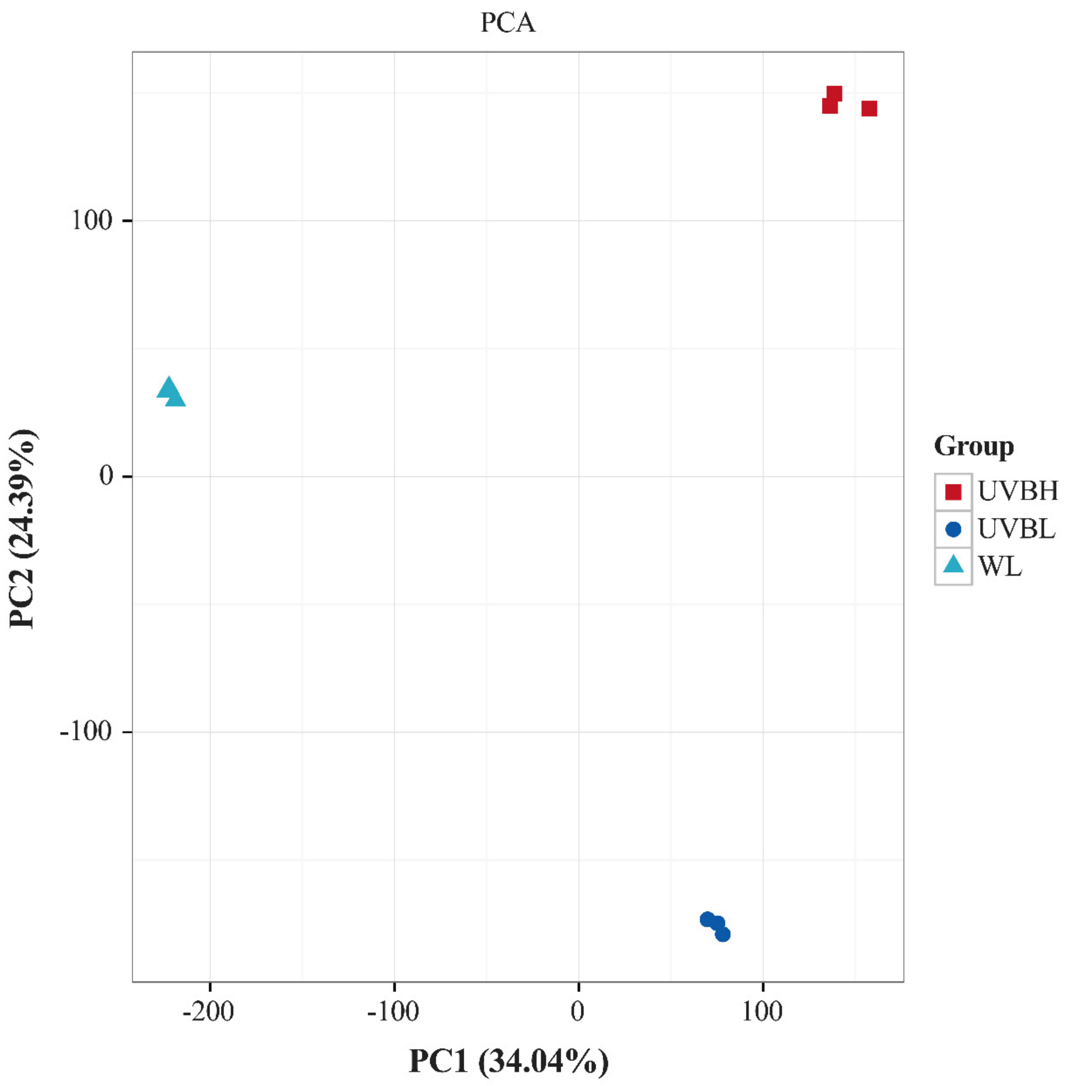

Figure S5. Principal component analysis plot. WL: white light, UVBL: white light supplemented with 0.1 W m^{-2} UV-B radiation, UVBH: white light supplemented with 0.4 W m^{-2} UV-B radiation.

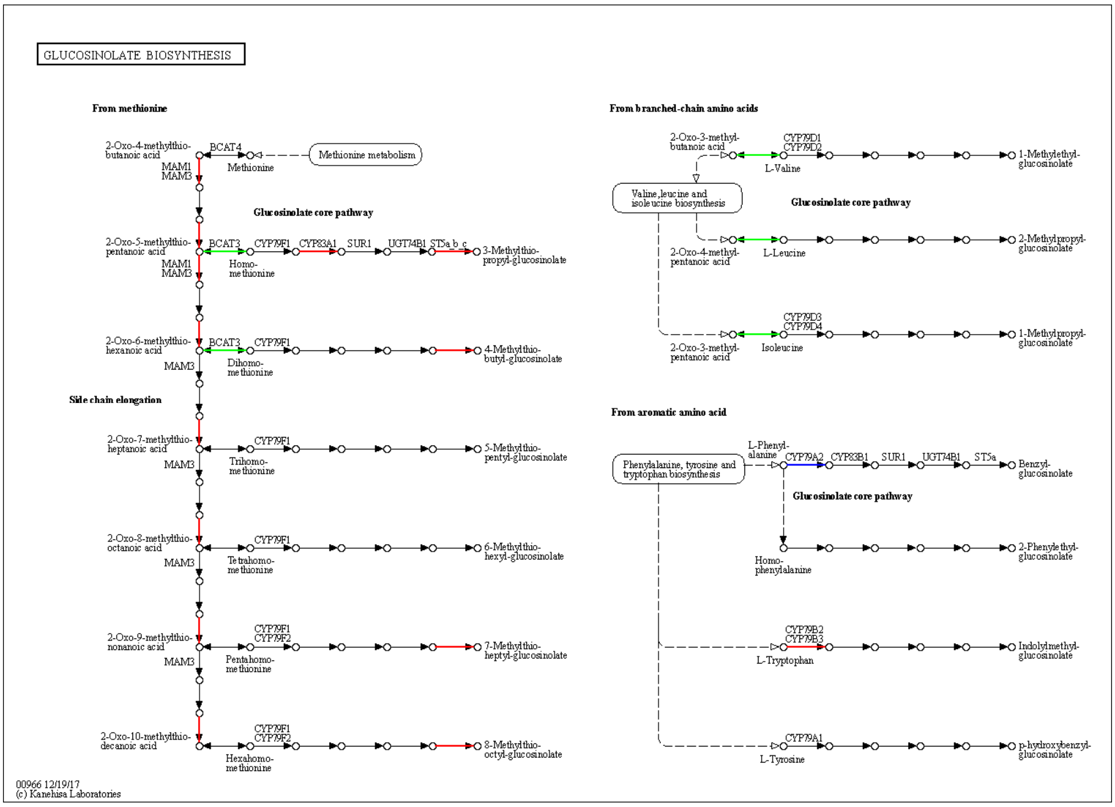

Figure S6. Glucosinolate biosynthesis of KEGG pathway enrichment analysis on DEGs in WL vs UVBL. Red arrows indicate upregulated expression, green arrows indicate downregulated expression, and blue arrows denote mixed expression changes.

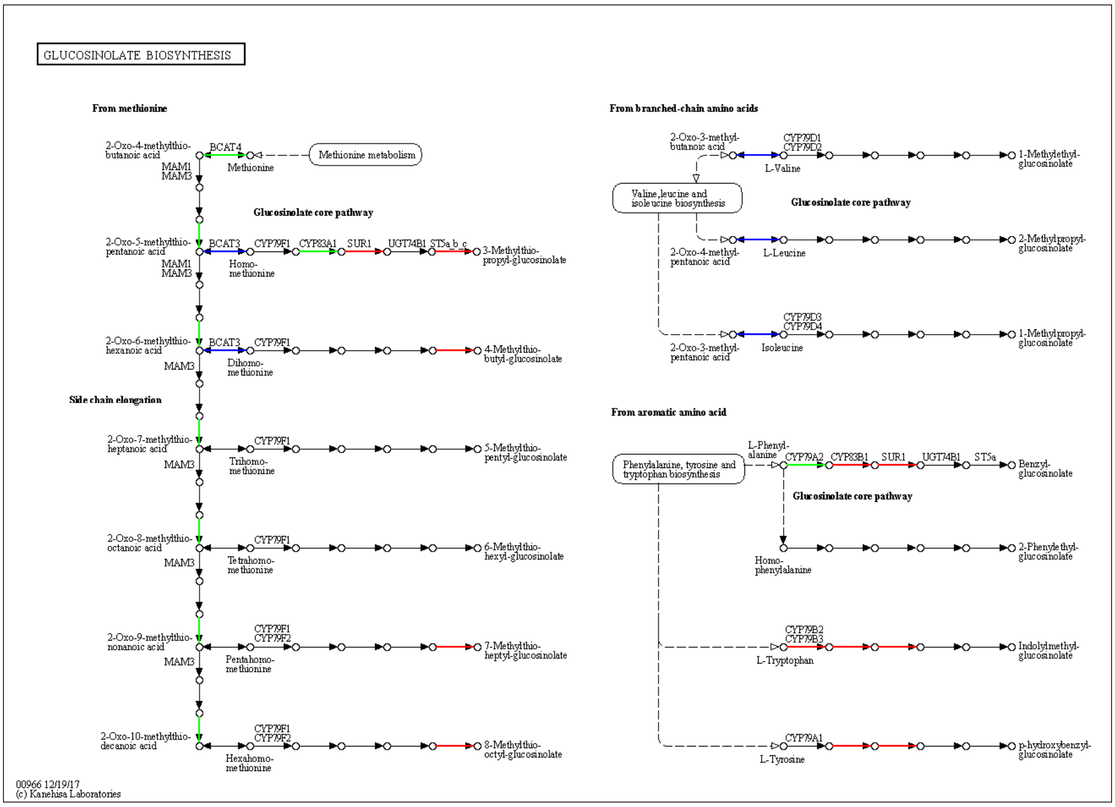

Figure S7. Glucosinolate biosynthesis of KEGG pathway enrichment analysis on DEGs in WL vs UVBH. Red arrows indicate upregulated expression, green arrows indicate downregulated expression, and blue arrows denote mixed expression changes.

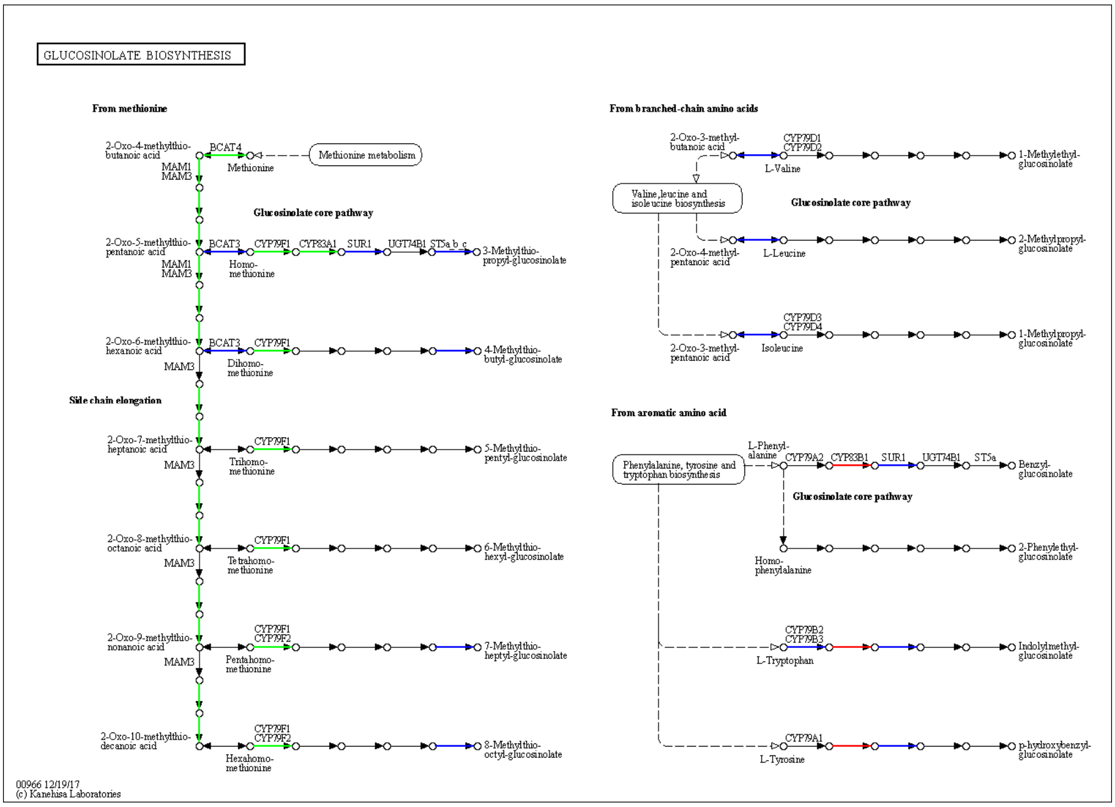

Figure S8. Glucosinolate biosynthesis of KEGG pathway enrichment analysis on DEGs in UVBL vs UVBH. Red arrows indicate upregulated expression, green arrows indicate downregulated expression, and blue arrows denote mixed expression changes.

Table S1. The primer sequences used in this RT-qPCR assay.

Gene ID	Encoded gene	Forward primer	Reverse primer
BnaA02G0394700ZS	*MYB28*	GCCCTACTGTCACCACCA	CGGAGGAAAGAGGACGAGAC
BnaA02G0391300ZS	*MYB34*	CGACCTTCACATTGCTCAA	CGGGAGATGACGACATATTG
BnaC05G0156000ZS	*MYB51*	CGTCGCCTCCATCCATGTT	GTTCTCAACACAAGACTCTTCCAAC
BnaC02G0280400ZS	*MYB122*	CAACAAATGGTCGGCGATAGC	GTGTCTCGGTATGGTCAGATGATT
BnaC07G0005900ZS	*SUR1*	CTGGATGGAAGATTGGCTGGAT	AGACTCTGCTTGATGGATTGAACC
BnaA09G0175100ZS	*SOT18*	GGAAGAGAAGGAAGAGGTTGTTGA	AGTTCTGCCAGTCTCCTACATTTC
BnaC03G0321100ZS	*AOP1*	GTGACAGCAAAGAAGACAAGGT	GAGCACAGAAAGCATGGAGAG
BnaA08G0186500ZS	*CYP81F4*	GACACTCAGGCTATATCCA	CTTCTCCTCCACCGTTAA
	Actin7	CTATCCTCCGTCTCGATCTCGC	CTTAGCCGTCTCCAGCTCTTGC

Table S2. Glucosinolate content of oilseed rape leaves in different light treatments. WL: white light, UVBL: white light supplemented with 0.1 W m^{-2} UV-B radiation, UVBH: white light supplemented with 0.4 W m^{-2} UV-B radiation. Data are expressed as average value ± SD (n=3) and significances between treatments at $P < 0.05$ are indicated by different lowercase letters.

Treatments	WL	UVBL	UVBH
Progoitrin	14.39±2.26 b	34.64±0.44 a	11.27±0.39 b
Glucobrassicanapin	2.25±0.07 a	1.37±0.12 b	0.38±0.10 c
Glucobrassicin	18.26±2.76 a	0.34±0.01 b	0.23±0.01 b
4-Hydroxyglucobrassicin	0.53±0.07 a	0.26±0.02 b	0.55±0.07 a
Neoglucobrassicin	3.35±0.13 b	29.34±0.72 a	0.25±0.00 c
Gluconasturtiin	1.53±0.23 b	0.37±0.01 b	8.32±1.26 a
Aliphatic glucosinolates	16.64±2.33 b	36.02±0.33 a	11.65±0.29 b
Indolic glucosinolates	22.14±2.71 b	29.94±0.72 a	1.03±0.06 c
Aromatic glucosinolates	1.53±0.23 b	0.37±0.01 b	8.32±1.26 a
Total glucosinolates	40.30±0.60 b	66.32±1.06 a	20.99±1.03 c

Table S3. The expression of DEGs involved in glucosinolate biosynthesis.

Gene symbol	Gene ID	Log$_2$FC			Annotation
		WL vs UVBL	WL vs UVBH	UVBL vs UVBH	
BCAT4	*BnaA03G0359600ZS*	0.848	—	—	methionine aminotransferase BCAT4 [Brassica napus]
	BnaA05G0362200ZS	-0.441	-1.624	—	BnaA05g36800D [Brassica napus]
	BnaC05G0396400ZS	0.106	-0.983	-1.085	methionine aminotransferase BCAT4 [Brassica napus]
	BnaC03G0438900ZS	-0.255	-1.946	-1.687	methionine aminotransferase BCAT4-like [Brassica napus]
MAM1	*NewGene_5983*	1.018	-0.438	-1.454	methylthioalkylmalate synthase 1, chloroplastic isoform X2 [Brassica rapa]
	NewGene_9944	0.591	-0.543	-1.132	unnamed protein product, partial [Brassica oleracea]
MAM2	*NewGene_9943*	0.793	-0.383	-1.173	methylthioalkylmalate synthase 2, chloroplastic-like [Brassica napus]
BCAT3	*BnaC01G0275700ZS*	-0.755	0.343	1.101	BnaCnng26670D [Brassica napus]
	BnaA01G0217100ZS	-0.120	0.195	0.319	branched-chain-amino-acid aminotransferase 3, chloroplastic-like [Brassica napus]
	BnaC08G0298700ZS	0.166	-0.252	-0.415	branched-chain-amino-acid aminotransferase 3, chloroplastic [Brassica napus]
IPMDH2	*BnaC04G0039400ZS*	0.521	-0.698	-1.217	PREDICTED: 3-isopropylmalate dehydratase small subunit 1 [Brassica oleracea var. oleracea]
IPMI2	*BnaA05G0036500ZS*	0.351	-0.532	-0.881	3-isopropylmalate dehydratase small subunit 1 [Brassica napus]
IPMDH3	*BnaC02G0060000ZS*	0.468	-1.436	-1.899	3-isopropylmalate dehydrogenase 3, chloroplastic isoform X1 [Brassica napus]
IMDH3	*BnaA02G0052200ZS*	1.264	-0.432	-1.693	3-isopropylmalate dehydrogenase 3, chloroplastic [Brassica napus]
CYP79F1	*BnaA06G0110600ZS*	0.959	-0.596	-1.551	BnaA06g11010D [Brassica napus]
	BnaC05G0136700ZS	0.754	-0.457	-1.208	BnaC05g12520D [Brassica napus]
CYP83A1	*BnaC04G0359300ZS*	0.637	-0.200	-0.835	cytochrome P450 83A1-like [Brassica napus]
	BnaA04G0267400ZS	0.590	-1.002	-1.589	cytochrome P450 83A1 [Brassica rapa]
GSTF11	*BnaA05G0489300ZS*	2.265	1.089	-1.172	glutathione S-transferase F11 [Brassica rapa]

Gene symbol	Gene ID	Log$_2$FC			Annotation
		WL vs UVBL	WL vs UVBH	UVBL vs UVBH	
	BnaC05G0546700ZS	0.848	-0.622	-1.467	BnaA05g32420D [Brassica napus]
SUR1	*BnaA09G0123400ZS*	0.529	0.013	-1.323	S-alkyl-thiohydroximate lyase SUR1-like [Brassica napus]
	BnaA07G0001300ZS	0.984	0.936	-0.045	S-alkyl-thiohydroximate lyase SUR1 [Brassica napus]
	BnaC07G0005900ZS	0.944	1.155	0.214	hypothetical protein DY000_02020094 [Brassica cretica]
UGT74B1	*BnaC05G0210800ZS*	0.779	0.414	-0.362	UDP-glycosyltransferase 74B1 [Brassica napus]
	BnaA09G0448300ZS	0.747	0.474	-0.270	UDP-glycosyltransferase 74B1 [Brassica napus]
UGT74C1	*BnaC04G0153200ZS*	1.159	0.226	-0.931	UDP-glycosyltransferase 74C1 isoform X1 [Brassica napus]
	BnaA05G0121200ZS	1.489	0.590	-0.896	UDP-glycosyltransferase 74C1 isoform X2 [Brassica napus]
SOT17	*BnaA06G0128100ZS*	1.836	0.869	-0.964	cytosolic sulfotransferase 17 [Brassica napus]
SOT18	*BnaC06G0404300ZS*	1.532	0.101	-1.430	cytosolic sulfotransferase 18 [Brassica napus]
FMOGS-OX2	*BnaA09G0155200ZS*	1.541	0.362	-1.177	flavin-containing monooxygenase FMO GS-OX2 [Brassica napus]
AOP1	*BnaC03G0321100ZS*	-0.201	-3.084	-2.878	probable 2-oxoglutarate-dependent dioxygenase AOP1 [Brassica oleracea var. oleracea]
GSL-OH	*BnaC03G0090500ZS*	1.380	0.033	-1.344	unnamed protein product [Brassica oleracea]
CYP79B3	*BnaA04G0145000ZS*	-0.575	1.042	1.619	hypothetical protein BRARA_D01320 [Brassica rapa]
	BnaC04G0436200ZS	—	3.656	2.351	PREDICTED: tryptophan N-monooxygenase 2 [Brassica oleracea var. oleracea]
CYP83B1	*BnaA08G0059700ZS*	0.323	1.363	1.042	hypothetical protein BRARA_H00529 [Brassica rapa]
	BnaC08G0083800ZS	0.242	1.226	0.987	PREDICTED: cytochrome P450 83B1 [Brassica oleracea var. oleracea]
GSTF9	*BnaC03G0173300ZS*	1.037	0.962	-0.073	glutathione S-transferase F9 [Brassica rapa]
GSTF10	*BnaC03G0173400ZS*	0.581	2.662	2.087	PREDICTED: glutathione S-transferase F10 [Brassica oleracea var. oleracea]
	BnaA03G0148900ZS	0.430	1.650	1.224	PREDICTED: glutathione S-transferase F10 [Brassica oleracea var. oleracea]

Gene symbol	Gene ID	Log2FC			Annotation
		WL vs UVBL	WL vs UVBH	UVBL vs UVBH	
SOT16	*BnaC02G0280500ZS*	-0.019	0.429	0.451	cytosolic sulfotransferase 16-like [Brassica napus]
CYP81F1	*BnaA01G0014700ZS*	-0.789	-1.326	-0.534	cytochrome P450 81F1-like [Brassica napus]
	BnaC01G0017200ZS	-0.485	-1.123	-0.636	cytochrome P450 81F1-like [Brassica napus]
CYP81F4	*BnaA08G0186500ZS*	-2.334	-3.966	—	cytochrome P450 81F4-like [Brassica rapa]
IGMT1	*BnaA02G0226700ZS*	0.818	-1.675	-0.855	indole glucosinolate O-methyltransferase 1 [Brassica napus]
IGMT2	*BnaA08G0244300ZS*	1.108	-1.889	-2.614	indole glucosinolate O-methyltransferase 2-like [Brassica napus]
CYP79A2	*BnaC09G0593900ZS*	-3.247	-1.482	—	phenylalanine N-monooxygenase [Brassica napus]
	BnaA10G0276300ZS	-4.075	-5.800	—	phenylalanine N-monooxygenase-like [Brassica napus]
CYP83A1	*BnaC04G0359300ZS*	0.637	-0.200	-0.835	cytochrome P450 83A1-like [Brassica napus]
	BnaA04G0267400ZS	0.590	-1.002	-1.589	cytochrome P450 83A1 [Brassica rapa]

Table S4. DEGs of transcription factor regulated glucosinolate biosynthesis.

Gene symbol	Gene ID	Log2FC (WL/UVBL)	regulated	Log2FC (WL/UVBH)	regulated	Log2FC (UVBL/UVBH)	regulated
MYB28	*BnaC02G0527500ZS*	1.254	up	0.637	normal	-0.613	normal
	BnaA02G0394700ZS	1.289	up	0.169	normal	-1.118	down
MYB34	*BnaC02G0523300ZS*	0.965	normal	-0.910	normal	-1.872	down
	BnaC07G0376700ZS	0.027	normal	-4.233	down	-4.257	down
	BnaA02G0391300ZS	1.561	up	-2.551	down	-1.751	down
MYB51	*BnaC05G0156000ZS*	0.195	normal	0.144	normal	-0.048	normal
	NewGene_10498	1.497	up	1.329	up	-0.165	normal

Gene symbol	Gene ID	Log2FC (WL/UVBL)	regulated	Log2FC (WL/UVBH)	regulated	Log2FC (UVBL/UVBH)	regulated
	NewGene_3075	1.853	up	1.364	up	-0.486	normal
	NewGene_1810	0.734	normal	0.699	normal	-0.031	normal
	NewGene_3965	1.700	up	2.510	up	0.811	normal
MYB122	*BnaC06G0278200ZS*	1.901	up	-1.028	down	-0.566	normal
	BnaC02G0280400ZS	1.369	up	0.743	normal	-0.343	normal
